# Safety and personalised care in maternity services of England for women whose preferred language is not English: a critical race theory analysis of interpreter experiences

**DOI:** 10.1136/bmjph-2025-003859

**Published:** 2026-03-31

**Authors:** Sarah Baz, Laura Sheard, Rachel E Rowe, Anne Macfarlane, Mashkura Begum, Wendy Olayiwola, Jennifer MacLellan

**Affiliations:** 1 Health Sciences, The University of Manchester, Manchester, England, UK; 2 NPEU, Nuffield Department of Population Health, University of Oxford, Oxford, UK; 3 School of Medicine, University of Limerick, Limerick, County Limerick, Ireland; 4 Public Contributor, Birmingham, UK; 5 National Maternity Lead for Equality, NHS England London, London, England, UK

**Keywords:** Community-Institutional Relations, Health Services Accessibility, Patient Harm, Public Health, Qualitative Research

## Abstract

**Introduction:**

Effective communication is central to safe, ethical maternity care. Women with cross-cultural communication needs are more likely to experience intersecting disadvantage, with poor maternal and infant outcomes. A professionally trained interpreter (PTI) can raise the quality of clinical care for patients to approach or equal that for patients without cross-cultural communication needs. The need to improve interpreter services has been recognised at the strategic level of the NHS to improve safety and personalised care. However, evidence for how to achieve this is limited from the UK context.

**Methods:**

We explored the experiences of PTIs working in maternity services in England using qualitative interviews. We analysed the data thematically, informed by Critical Race Theory, which argues that inequality is deeply embedded in policy, law and institutional structures and practices. We discussed interim findings with our lived experience group.

**Results:**

We interviewed 28 interpreters with a range of qualifications who worked for language agencies or in-house NHS interpreting services. Our analysis constructed three themes: The ‘shady’ agency; ‘You can get anyone and you don’t know how experienced they are’; ‘you are never part of a team’. We found that institutional practices and outsourcing marginalise interpreters, compromising worker well-being and patient safety.

**Conclusion:**

To ensure patient safety, it is essential for the NHS to recognise the professional status of medical interpreters and integrate PTIs into the core clinical team. This will require investment in standardised interpreter training, access to supervision and career development. Embedding interpreters in NHS safety culture is essential for equitable and effective care.

**WHAT IS ALREADY KNOWN ON THIS TOPIC**

- Absent or ad hoc professionally trained interpreters in maternity care act as an independent risk factor for poor outcomes and contribute to disparities in maternal mortality.

**WHAT THIS STUDY ADDS**

- This study used Critical Race Theory to expose how the current systems used by language agencies to fulfil assignments, monitor and support interpreters can compromise safety in an interpreted consultation and reinforce inequity.

**HOW THIS STUDY MIGHT AFFECT RESEARCH, PRACTICE OR POLICY**

- To ensure patient safety and equity, it is essential for language agencies and healthcare organisations to implement standardised training, support, evaluation and appropriate remuneration for interpreters.

## Introduction

 The WHO advocates for women’s values and preferences to be at the centre of their care during pregnancy, childbirth and the postnatal period.^1^ The National Health Service (NHS) in the UK is committed to everyone using maternity services to receive safe, personalised care that is centred around the unique needs and circumstances of each family, with women having choice about the care they receive, informed by impartial information.^2^ Effective communication is central to safe, ethical maternity care.^3^ Women with cross-cultural communication needs (where linguistic and cultural background are different to those of the health provider) are more likely to experience intersecting disadvantage, with poor maternal and infant outcomes.^4^ Thus, it is imperative that they are able to ask questions, understand the answers, be aware of their rights and know where to seek help.^5^ Women with cross-cultural communication needs are at risk of inadequate, unconsented care, leaving them feeling isolated, unsupported and unable to communicate.^46^,^8^

A professional trained interpreter (PTI) is an interpreter with training in interpreting skills who is fluent in the spoken and translated language. Their use can raise the quality of clinical care for patients to approach or equal that for patients without cross-cultural communication needs.^9^ PTIs view their work as integral to the delivery of safe care for patients.^10^ Evidence suggests that PTIs are underused in maternity practice, with high incidence of ad hoc and informal interpreters, such as family and friends, which is not recommended,^11^ or community interpreters who have varying levels of training or qualification.^12^ A woman’s need for an interpreter is not consistently documented across NHS maternity services.^12^ From limited information available (from a freedom of information request across England from 2020 to 2022), women with a documented need for an interpreter received an average of only three interpreted sessions across a minimum recommended 17 contacts for a first-time mother in their maternity journey.^12^ Dissatisfaction with interpreter provision among service users and healthcare professionals includes reports of poor professional practice compromising confidentiality, disclosure and accuracy.^13^,^15^ When PTIs are not used when needed, this is an independent risk factor for poor outcomes,^516^,^18^ unconsented procedures (such as episiotomy in labour),^19^ birth trauma^6 7^ and contributes to disparities in maternal mortality.^20^

The need to improve PTI services has been recognised at the strategic level of the NHS to improve safety and personalised care.^15^ However, evidence for how to achieve this is limited from the UK context. Most interpreting services have been out-sourced to language agencies since 2015 with little publicly available information about their processes and standards. There are only a few in-house NHS interpreting services that have re-established themselves in a hybrid model with the contracted agency, in response to specific local circumstances. There are two studies from more than 10 years ago to suggest that medical interpreters find the agency interpreting model to be challenging. They report a lack of information about assignments or preparation with specific medical terminology, lack of training and support and incompatible rewards,^16 21^ but there is no current evidence that unpacks the challenges experienced by interpreters in the different models of service provision and how this may impact patient care, and none specifically relating to maternity care. In this paper, we aim to explore the experiences of PTIs within the current systems for interpreter provision in English maternity services, through a patient safety lens.

## Methods

### Study design

This qualitative interview study was a component of a larger study using participatory approaches and implementation science to optimise the use of PTIs in maternity services of England (NIHR157976).^22^

In this paper, the analysis was informed by Critical Race Theory (CRT), which argues that racism is deeply embedded in policy, law and institutional practices. Structural racism perpetuates racial inequalities and intersects with other identities to shape how individuals experience oppression.^23^ This is evidenced by, for example, ethnic minority workers consistently experiencing lower pay than white British workers,^24^ being over-represented in low-paid unstable jobs^25^ and experiencing fewer opportunities for promotion or career progression despite having qualifications—all of which are linked to the impact of lower social capital within the workplace.^26^ These racial experiences intersect with characteristics of gender and older age to amplify disparities in employment pay and conditions.^27 28^ These racial and structural inequalities have not been examined in relation to the specifics of interpreting services and their impact on patient safety in NHS maternity care.

### Participants and sampling

We used a combination of purposive and snowball sampling between January and May 2025 to recruit interpreters, managers of in-person interpreting services and commercial agency interpreting service providers who had experience of interpreting within maternity care in England within the last 12 months. Interpreters were identified through online publicly available interpreter directories, professional and personal contacts of the research team and snowball sampling.^29^

Eligible participants were emailed with brief details of the study and invited to contact the study researcher if they wished to participate in an interview or focus group discussion. Respondents were sent an information sheet about the study. Those who agreed to take part gave informed verbal consent before being interviewed. All participants preferred individual interviews for convenience and to maintain confidentiality. We purposively sampled for diverse experiences to clarify, check and confirm our findings, particularly between ‘in-house’ and ‘agency’ employed interpreters as they reported different working conditions. We collected participant characteristics of language interpreted, geographic location, years working as an interpreter and qualifications in interpreting to ensure variation in our sample. We contacted interpreting managers of in-house interpreting models and representatives of language agencies for interview to gain a strategic view that would contextualise and inform the operational experiences shared by interpreter participants.

### Data collection

A topic guide was developed to elicit an interpreter’s perspective on the study research question: what are the barriers and facilitators to the use of PTIs in maternity care. The guide was checked with six women with lived experience of interpreting during maternity care, in the study Patient and Public Involvement and Engagement (PPIE) group. The guide used open questions, creating opportunities for the participant to develop and expand on subjects most relevant to their experience (online supplemental file 1). Data were collected through single episode interviews, in a private space, with informed consent recorded. All interviews were conducted online or by telephone, except for one who was interviewed in person, by SB, an experienced post-doctoral, multi-lingual qualitative researcher. The researcher had no prior relationship with the participants. Audio recordings were transcribed verbatim, checked against the original recording and loaded into the data analysis software, NVivo 15.^30^

### Data analysis

Our sensitising concept for this analysis was CRT (as described in the Study design section above) with the aim of moving from describing ‘what happens’ to exploring how racialised systems of power produce what happens. We employed thematic analysis as our approach to generating inductive codes and themes from the data.^31^ Transcripts were read repeatedly by two researchers (SB and JM), remaining attentive to systems, hierarchies and institutional processes, interrogating the data for: mechanisms of power; whose voices are marginalised; and how intersecting identities shape the participants’ experiences.^23^ Line-by-line coding was conducted by the primary researcher, staying close to participants’ words. The two researchers discussed the emerging codebook and reflexive memos in regular analysis meetings with the wider research team. We interpreted patterns using CRT concepts, ensuring themes reflected systemic processes rather than individual experiences. Sampling continued until additional interviews were not generating new understanding in the data.^32^

### **Patient** and public involvement

We shared our interim findings with our PPIE group. This offered an opportunity to integrate multiple perspectives into the research cycle and support the identification of contextually informed research findings.^33^ They shared personal examples related to structural influences on interpreting support, and both shared and differential features of interpreting across agency and in-house providers that resonated with our interpretation of the interview data.

### Reflexivity

Reflexivity was integral to this study as knowledge production is shaped by researchers’ social positions, professional backgrounds and relationships to the research topic. The primary researcher is multilingual and from a minoritised ethnic background, bringing lived and professional insight into the data collection and analysis. This positionality informed sensitivity to participants’ accounts while also requiring ongoing reflexive attention to potential assumptions or over-identification.

The wider multidisciplinary research team brought complementary expertise in maternity, qualitative methods and health inequalities, providing both insider and outsider perspectives. Reflexive practices, including memo writing and regular analytic discussions, were used to critically examine interpretations and situate participants’ experiences within structural and institutional contexts rather than individualised explanations. This reflexive approach supported an analysis foregrounding racialised power relations, institutional accountability and interpreters’ experiential knowledge, in keeping with CRT principles.

## Results

We interviewed 28 interpreters, including one bi-lingual healthcare professional and two people at managerial level from in-house interpreting services (one with experience of interpreting, one managing an interpreting service). No agency providers responded to our invitation to interview. Interview discussions lasted between 29 and 91 min (average 50 min). Selected participant characteristics are summarised in table 1. Interpreters spoke a range of languages including Portuguese, Urdu, Dari, Arabic, Sylheti, Sorani, Ukrainian and Tigrinya. Participants completed both in-person and remote interpreting assignments across England, regardless of their base location.

**Table 1:** Participant characteristics

Demographics	No	%
Professional role		
Antenatal classes interpreter	2	7
Language agency	14	50
In-house interpreter	9	32
Healthcare professional	1	4
Interpreting manager	2	7
No of years working professionally		
0–5 yrs	5	18
6–10 yrs	8	28
11–15 yrs	1	4
16–20 yrs	3	11
21–25 yrs	8	28
26–30 yrs	1	4
30+yrs	2	7
Qualifications		
None	6	21
Level 1	2	7
Level 2	1	4
Level 3	12	42
Level 4	1	4
Level 6	1	4
Level 7	2	7
Other	3	11
Age		
Did not disclose	3	11
21–30	3	11
31–40	3	11
41–50	11	39
51–60	6	21
More than 60	2	7
Gender		
Male	2	7
Female	26	93

### Context of maternity interpreting services in England

PTI services for hospital based maternity care are largely provided by language agencies, who compete for contracts with the NHS (their ‘client’) to provide in-person and remote interpreting services. Interpreters are self-employed, typically register with several agencies and are then given assignments. These can be in education and the police service as well as the NHS. Participants described how some hospitals continue to have or have introduced in-house interpreters, while others have ‘link workers’ who provide an ‘advocacy’ role for pregnant women. Participants described providing cross-cultural and language support for pregnant women at every stage of the maternity journey.

Three inter-linked themes were identified from our analysis: The ‘shady’ agency: ‘I describe it as serfdom’; ‘You can get anyone and you don’t know how experienced they are’; ‘you’re never part of a team’.

#### The *‘shady’* agency: ‘I describe it as serfdom’

Participants’ accounts frame agency interpreting as a highly transactional labour arrangement embedded within institutional structures that marginalise linguistically minoritised service users while simultaneously producing conditions of culturally unsafe care. Some participants valued the flexibility of agency-based interpreting, particularly telephone interpreting, as it was compatible with other life commitments. However, this flexibility operated within a system of constrained choice shaped by unequal hierarchies. Participants described systematic commodification of interpreting labour with systemic problems including poor pay and restrictive working conditions that contribute to social and economic inequalities. One participant, who was a qualified interpreter with 15–20 years’ experience, described having to decline an assignment due to its ‘low rates’ (Interpreter 4). She described how the agency would then approach an interpreter with less experience and qualifications to fill the assignment at a lower cost. Such arrangements shift institutional risk onto interpreters and families, while obscuring responsibility for the quality and safety of cross-cultural healthcare encounters. Our participants’ accounts describe an environment in which agencies undervalue expertise, with a greater focus on cost efficiency than on equitable or ethical practice. Such practices undermine professional standards and increase the risk of culturally unsafe interactions, as patients and clinicians are deprived of interpreters equipped to support nuanced, respectful and contextually informed communication. Participants’ accounts positioned agencies as powerful intermediaries that extract value from racialised labour while remaining largely unaccountable for the cultural safety or ethical quality of care delivered. The absence of effective feedback or governance mechanisms between agencies and NHS organisations was understood as enabling these inequities to persist, reinforcing a system driven by profit rather than patient safety or equity:

They [agencies] recruit individuals who have no qualifications, who are unaware of the code and conduct of the interpreters. So, someone could speak an average level of English and they would be deployed to cover such bookings for such rates (Interpreter 4, 15–20 years experience)

Participants described having to assess the financial viability of an assignment in a context where preparation work is not paid, mileage for in-person interpreting was often capped and parking was not reimbursed. Refusing assignments on financial grounds was perceived as incurring punitive consequences, including reduced access to future assignments from the agency. This results in coerced compliance where interpreters felt compelled to accept conditions that compromise both their well-being and the cultural safety of the interpreting encounter. Structural racism operates here through normalised precarity framed as ‘flexibility’, aligning with gendered expectations that women accommodate caregiving responsibilities, with age and migration status interacting to structure vulnerability differently across the workforce. Interpreter 16 described how they would answer all calls from the agency even if it was not convenient:

But sometimes there is a call directly from the agency, so it means that they couldn’t find an interpreter. So in these situations, I… I would always pick up, yeah, but even if I am on a street or in the shop…I would stop somewhere in front of the shelves in the shop, stop and interpret. (Interpreter 16, 0–5 years experience)

This meant that she might not be in a private location during the consultation, raising concerns about confidentiality, trust and patient dignity, which are all components of culturally safe care. Such practice places significant cognitive and emotional strain on the interpreter as they try to maintain professionalism and accuracy in an environment that may be noisy, or constitute a public space. Such experiences reinforce a model of care in which the needs of linguistically marginalised families are subordinated to institutional efficiency.

Interpreter 10 described receiving 27 pence per minute for telephone interpreting while being aware that the agency charges the client (the NHS) £2 per minute. When they tried to negotiate a higher rate, they were removed from the pool of interpreters. The appointment went unfulfilled. This withdrawal of work functioned as a disciplinary mechanism, silencing interpreters’ voices and reflecting structural devaluation of age and experience.

I describe it as ‘serfdom’. In (medieval times) most contracts of serfdom, he will produce … whatever was possible from the land…and the huge percentage of this will go to the lord because of land fees, equipment fees, accommodation fees. (Interpreter 10, 5–10 years experience)

Participants identified how a lack of meaningful feedback systems or collective voice allowed these practices to continue unchallenged. Viewed through a CRT lens, these findings demonstrate how poor pay, structural dependency, interpreter precarity and systemic lack of accountability sustain a model of care that is exploitative of interpreters and culturally unsafe for minoritised families.

#### ‘You can get anyone and you don’t know how experienced they are’

Participants explained how there was no minimum standard of education or subject specific experience to work as an interpreter in a maternity setting. From a CRT perspective, this absence of agreed standards reflects a structural devaluation of linguistic and cultural labour, positioning interpreting as a natural linguistic ability rather than a professional skilled, professional expertise. Participants described this situation as institutional disregard for the safety and quality of care provided to women with cross-cultural communication needs. Rather than being treated as a specialist clinical role integral to equitable maternity care, interpreting was positioned as a low-skilled, transactional service. This devaluation was closely linked to pay structures that failed to recognise qualifications or experience. One participant with 20–25 years’ experience described how remuneration was the same across qualification levels, while rates had steadily declined over time:

the pay offered is exactly the same to people who have a Level 3 Community Interpreting or a Level 6…each time a new company takes over, they’re paying less and less to the interpreters. So then they’re encouraging unqualified people to come, because people who are generally qualified will feel disheartened by being paid less each year than they were. I actually earn less now than I did 20 years ago. (Interpreter 9, 20–25 yrs experience)

From a CRT perspective, this illustrates how commissioning practices reproduce racialised labour hierarchies, incentivising the recruitment of less qualified, more economically precarious interpreters while displacing experienced practitioners. Such practices function as a form of institutional racism, normalising the erosion of professional standards in services predominantly used by racially and linguistically minoritised women.

As shown in table 1, participants reported a wide range of qualifications and experience in interpreting. While some gained their experience from ‘just working’ (Interpreter 20), others emphasised how formal training often failed to prepare them for the realities of maternity care, with much learning achieved ‘on the go’ (Interpreter 13). Participants across the sample consistently described a lack of ongoing support through mentoring, appraisal or structured professional development. Interpreter 10, employed through an in-house ‘staff bank’ on a zero hours contract, highlighted the absence of institutional responsibility for maintaining professional standards:

there’s no professional development, there’s no appraisal, there’s no sit down, come and talk. I know we’re still bank staff, but because we’re in house, there needs to be… you know, just to make sure everybody’s performing to a certain standard and conduct of professionalism. (Interpreter 10, 5–10 years experience)

Agency-based participants described being categorised as ‘freelance’, with agencies acting as brokers rather than employers. This arrangement transfers responsibility for qualifications, preparation, professional conduct and development entirely on the interpreter. If the appointment did not go well, or a mistake was made, participants reported that the agency was not held accountable, with responsibility individualised and placed on the interpreter. From a CRT perspective, this reflects a racialised hierarchy of risk in which marginalised workers bear the consequences of institutional failure, without access to protections, recognition or voice.

Participants emphasised that knowledge of the health and maternity system, gained from prior qualifications or years of experience, was essential for patient safety, yet this expertise was not required or formally recognised by agency employers:

If you have some knowledge it would be easier to prevent information to be missed, like miscommunication (Interpreter 15, 5–10 yrs experience)

CRT highlights how experiential and contextual knowledge constitutes racialised epistemic labour that is systematically undervalued, despite being a core component of safe, equitable care. This lack of recognition increases the risk of miscommunication in maternity consultations, disproportionately affecting racially minoritised women. Participants described receiving insufficient information about the focus of an appointment, limiting their ability to prepare for emotionally complex or technically demanding consultations. Assignments could involve antenatal screening, discussions about genetics and risk, birth preferences or breaking sad news. Interpreter 15 described using AI to generate common medical terms while travelling to the assignment. This highlights how interpreters compensate for institutional gaps in support. Participants identified simple interventions, such as glossaries or briefing proformas, as ways to improve safety and quality:

there is a lot of terminology that could be created as a starting point for those starting out…there could be a set of a glossary with explanation, and then the interpreter gets such a glossary and only interpret that into their own language (Interpreter 10, 5–10 years experience)

Participants described a system in which interpreters’ work, expertise and well-being are largely invisible, and navigation of clinical encounters without adequate support and preparation is normalised. A lack of preparation for breaking sad news was described as particularly difficult. Interpreter 7 described such situations as like entering ‘*a dark room*’. When she contacted the agency to request more information prior to an assignment in the future, she was told that this responsibility lies with the hospital rather than the agency. This illustrates how accountability is routinely displaced across institutions. Interpreter 3 stated how he would try to ask for a briefing from the clinicians on arrival. This was easier in a face-to-face appointment:

I would have everything explained in advance if it is a serious matter…. So the specialist, either the nurse or a consultant, they would quickly brief me prior to the actual appointment with the patient so that I know how to present myself. (Interpreter 3, 5–10 years experience)

These findings indicate significant variability in interpreters’ training, experience and knowledge of the clinical area. There is a systemic absence of supervision, regulation, career development or appraisal for PTIs. Viewed through a CRT lens, this lack of oversight by the commissioning body and/or the health service reflects the de-prioritisation of services used by racially and linguistically minoritised women. These structural conditions risk compromising patient safety and equitable maternity care and reproduce institutional equities that position interpreters as dispensable rather than integral to high quality healthcare delivery.

#### ‘You’re never part of a team’

Many of the agency interpreters described interpreting as being a ‘lonely’ profession not merely due to the nature of the job, but because of systemic conditions that actively isolate them. Participants described being ‘thrown into the deep end’ without access to mentoring or peer support and working alone with no ‘community’ of interpreters or sense of belonging. Participants reported how little support was available through the agency to debrief when encountering emotional topics:

I have to be very professional and not too emotionally involved. But as a human, how can you not be emotionally involved?… it’s all very internal as an interpreter… you’re very lonely to be honest, ’cause you’re never part of a team. (Interpreter 18, 20–25 years experience)

Interpreters described working in a system in which they were expected to absorb the emotional labour of frontline healthcare without acknowledgement or protection, reinforcing broader patterns of invisibilised work and structural inequity within the healthcare system. Interpreter 26 described the theoretical availability of debriefing via email with the agency, but she had never used this option as it did not feel safe or appropriate to process emotionally complex experiences through the impersonal medium of email. This places the support mechanisms as symbolic compliance rather than meaningful care, further isolating interpreters. In contrast, in-house interpreters described being in a more structurally supportive environment, part of a team, with emotional and peer support available:

We’ve got a nice manager [who is also an interpreter] who understands us… and every, say, four weeks, five weeks, we’d all get together, and we’d talk about what we’ve gone through… so you’re talking to somebody that’s got experience into that sort of thing. (Interpreter 22, 25–30 years experience)

The ability to offer continuity of support to families was described as a meaningful aspect of PTI work, enabling the development of trust, cultural connection and sustained engagement in the care journey. Often, the in-house interpreters knew families and women for years and described feeling ‘more connected’ than when doing agency work. They would follow their care to the Neonatal Intensive Care Unit and the paediatric department, supporting families and ensuring small details were not overlooked. However, this relational continuity is frequently undermined by institutional systems as feminised and based on gendered expectations of availability and care. This was seen in agency booking protocols that prioritise efficiency over relational care. One participant described how she had supported a woman throughout her pregnancy but was excluded from the planned caesarean birth:

she told me that on the day of the C-section that they had forgotten to book me as the interpreter and then booked somebody at short notice that was a total random stranger to her (Interpreter 9, 20–25 years experience)

From a CRT perspective, this example illustrates how bureaucratic systems reproduce culturally unsafe care, treating language support as a transactional task rather than relational and undermining trust at critical moments.

Some participants described encouraging women to explicitly request the same interpreter for subsequent appointments:

Please book this interpreter for the next time as well because I’m very comfortable with her and she knows everything (Interpreter 2).

While some agencies booked the same interpreters, others did not. With the language agency being in control of the booking system, participants’ ability to maintain continuity was severely constrained. Support across appointments was described as easier to facilitate for in-house interpreting staff as they knew the hospital procedures and staff, were part of the woman’s care pathway and could work with them to be present at their appointments. Interpreter 22 described ‘it’s not just interpreting and then go’, you can ‘follow it through’ along the pregnancy journey. Interpreter 22 described supporting a consultation where the pregnant woman’s partner was making her birthing choice for her. In this instance, the interpreter was able to raise a concern with the healthcare professional and, through collaborative planning, created space for the pregnant woman to express her own wishes. The woman’s decision to pursue a normal birth, contrary to her husband’s directive, and her expression of gratitude ‘I’m so grateful. Thank you so much for giving me the power to make my decision’ highlighted how language access can function as a vehicle for reclaiming bodily autonomy and decision-making power in healthcare encounters.

CRT highlights how such moments of agency are significant for racially minoritised women, whose voices are often marginalised within clinical settings. Yet such relational interpreting is fragile and undermined by agency models that fragment care and isolate interpreters. These data show the excessive professional responsibility on interpreters and the isolation they experience to be systemic problems that undermine their professional experience, well-being and the quality of the interpreting they can provide.

## Discussion

Our analysis has elucidated the ways in which structural and organisational level factors impact PTIs and patient safety, equity and quality of care for those who need interpreted consultations. Our findings report how poor pay, working conditions and a lack of standardised training for interpreters working in maternity services act to undermine PTI professionalism and directly impact patient safety.

The concept of professionalism in healthcare is institutionally defined by regulatory standards, certification and trust in a consistent level of competence.^34^ However, public service interpreting—including medical interpreting—remains outside this framework, lacking mandated regulatory oversight or standardised qualifications.^11 35^ This regulatory vacuum systematically undermines the professional standing of PTIs and patient safety. Our findings reveal significant variability in interpreter qualifications and experience, shaped by low remuneration, fragmented employment structures and enabled by contracting practices that obscure accountability.^34^ Our study reports how PTIs are most commonly hired from a language agency. Outsourcing NHS functions has been described in other contexts (eg, cleaners and porters) as creating a two-tier system, structurally excluding staff from NHS safety culture.^36^,^38^ The in-house interpreters within our study reported greater integration within the hospital system, but since they worked across clinical areas, they were not always considered part of the maternity clinical team. This structural exclusion reflects a system level division of work that renders interpreter contributions invisible and devalues their role in safe and equitable care delivery, denying them the institutional recognition, support and resources afforded to NHS-employed staff.

Inclusive working environments that promote psychological safety typically achieve the best outcomes for patient safety.^39^ Participants who were agency-employed reported working in professional isolation without pre-assignment briefings, continuity or debriefing opportunities to reflect on consultations and identify improvements. Debriefing is known to enhance care quality, safety culture and practitioner well-being, yet remains underused in clinical settings, as our data also shows.^40 41^ This lack of feedback and reflection undermines learning and disregards the emotional labour involved in the work for both interpreters and healthcare professionals in cross-cultural consultations. The fact that the interpreter is described in the professional literature as independent within the consultation and thus protected from emotional harm^34^ reinforces the transactional classification of interpreting work within healthcare and shields the institution from responsibility for their well-being. This framing reinforces their transactional status, limits their inclusion in care quality and safety strategies and constitutes a structural patient safety risk.

The positive impact of continuity of carer within maternity is well-established.^42 43^ Our study supports this evidence base to show how continuity of interpreter may hold similar benefits, especially during high-risk moments that blend physical and emotional vulnerability.^34^ Interpreters who are familiar with patients and are culturally and linguistically congruent are uniquely positioned to promote a sense of safety and prevent emotional harm.^44 45^ However, the participants in our study described a system where the transformative experience of interpreter continuity is secondary to bureaucratic convenience. The inflexibility of language agency systems within clinical care structures is known and does not appear to have improved since first reported more than ten years ago.^46 47^ This stagnation contributes to interpreter frustration, low morale and threats to professional retention in health service interpreting.^48^ The continued marginalisation of interpreters—through lack of structural support, professional recognition and integration into safety cultures—perpetuates systemic inequities. These conditions impact not only interpreter well-being and job satisfaction but also the emotional and physical safety of women and families from minoritised backgrounds.^32 48^ By disregarding the interpreter role as central to safe, culturally competent care, current systems risk sustaining structural harm within healthcare delivery.

## Limitations

This study has revealed valuable insights into maternity interpreting in the UK context. The self-selecting sample of 28 interpreters has provided rich experiences but may limit transferability of our findings to other contexts. The limited participation of interpreting managers suggests their perspectives have not been as fully explored as that of the interpreters. The use of individual interview over focus groups, as originally planned, removes the opportunity to analyse group dynamic and collective meaning making. Despite these limitations, the study’s strength lies in the nuanced insights grounded directly in the interpreters’ experience.

## Conclusion

To ensure patient safety, it is essential for the NHS to recognise the professional status of medical interpreters and integrate PTIs into the core clinical team. This will require investment in and regulation of standardised interpreter training, with access to supervision, career development and reflective practice. Systems that support continuity of interpreter will enable the building of trust and mitigate against harm. This will ultimately impact safety in care. Applying a CRT lens to our analysis has revealed how institutional practices and outsourcing marginalise racialised and multilingual professionals, compromising both worker well-being and patient safety. Embedding interpreters in the NHS safety culture is essential to achieve equitable and effective care.

## Supplementary material

10.1136/bmjph-2025-003859 online supplemental file 1

## Data Availability

Data are available upon reasonable request.
