# Supplementary material for: Safety and personalised care in maternity services of England for women whose preferred language is not English: a critical race theory analysis of interpreter experiences
Source: BMJ Public Health. 2026 Mar 31;4(1):e003859. doi: 10.1136/bmjph-2025-003859 (PMC13052530; doi:10.1136/bmjph-2025-003859)
Supplement: online supplemental file 1 [file bmjph-4-1-s001.docx]

**OPTIM-I: Optimising the use of professionally trained interpreters in maternity services of the UK**

**Interpreter Interview Schedule**

The OPTIM-I study is aiming to improve the quality and safety of cross cultural consultations by learning from maternity practitioners and interpreters about how they conduct these consultations, what works and what’s difficult, and what support is needed to help provide the best care for women and babies. We are recruiting up to 30 interpreters from across England who have experience of interpreting in maternity, to take part in interviews about their experiences for this study. All experiences will be anonymised and looked at together. We hope to identify and summarise what supports or hinders interpreters in cross cultural consultations in maternity. We will present these findings to small groups of practitioners, interpreters and women in the co-design component of the study where we will work together to create solutions and ways to share best practice. The interview should last about 30-40 minutes, you can choose not to answer any of the questions, and pause or stop at any time. We will go through the consent form before we start.

1. Can I start by asking you to share how many years you’ve worked as an interpreter and what areas have you worked in?
2. What training or preparation have you had in doing interpreting work in maternity settings?
   1. breaking bad news,
   2. seeking consent,
   3. presenting choice,
   4. speciality training in maternity vocabulary or
   5. maternity pathway navigation
3. What accreditation or certification/professional development is available to you as an interpreter?
4. Can you describe the role of cultural navigation in your interpreting work?
5. What support systems are offered in your organisation for you as an interpreter (briefing and debriefing/psychological support)
6. From the experience of yourself or your colleagues, can you share any challenges an interpreter may face when conducting a cross cultural consultation?
7. Do you have any recommendations of how practitioners (maternity or interpreters) could be best supported to conduct a cross cultural consultation?
